# A modified formula using energy system contributions to calculate pure maximal rate of lactate accumulation during a maximal sprint cycling test

**DOI:** 10.3389/fphys.2023.1147321

**Published:** 2023-04-13

**Authors:** Woo-Hwi Yang, So-Young Park, Taenam Kim, Hyung-Jin Jeon, Oliver Heine, Sebastian Gehlert

**Affiliations:** ^1^ Graduate School of Sports Medicine, CHA University, Pocheon-si, Gyeonggi-do, Republic of Korea; ^2^ Department of Medicine, General Graduate School, CHA University, Pocheon-si, Gyeonggi-do, Republic of Korea; ^3^ Olympic Base Center Rhineland, Cologne, Germany; ^4^ Department for Biosciences of Sports, Institute of Sports Science, University of Hildesheim, Hildesheim, Germany; ^5^ Institute of Cardiovascular Research and Sports Medicine, German Sport University Cologne, Cologne, Germany

**Keywords:** anaerobic performance, diagnostics, glycolytic metabolism, lactate, anaerobic power output

## Abstract

**Purpose:** This study aimed at comparing previous calculating formulas of maximal lactate accumulation rate (^*ν*^_La.max_) and a modified formula of pure ^*ν*^_La.max_ (P^*ν*^_La.max_) during a 15-s all-out sprint cycling test (ASCT) to analyze their relationships.

**Methods:** Thirty male national-level track cyclists participated in this study (*n* = 30) and performed a 15-s ASCT. The anaerobic power output (W_peak_ and W_mean_), oxygen uptake, and blood lactate concentrations (La^−^) were measured. These parameters were used for different calculations of ^*ν*^_La.max_ and three energy contributions (phosphagen, *W*_PCr_; glycolytic, *W*_Gly_; and oxidative, *W*_Oxi_). The P^*ν*^_La.max_ calculation considered delta La^−^, time until W_peak_ (t_PCr−peak_), and the time contributed by the oxidative system (t_Oxi_). Other ^*ν*^_La.max_ levels without t_Oxi_ were calculated using decreasing time by 3.5% from W_peak_ (t_PCr −3.5%_) and t_PCr−peak_.

**Results:** The absolute and relative *W*_PCr_ were higher than *W*_Gly_ and *W*_Oxi_ (*p* < 0.0001, respectively), and the absolute and relative *W*_Gly_ were significantly higher than *W*_Oxi_ (*p* < 0.0001, respectively); ^*ν*^_La.max_ (t_PCr −3.5%_) was significantly higher than P^*ν*^_La.max_ and ^*ν*^_La.max_ (t_PCr−peak_), while ^*ν*^_La.max_ (t_PCr−peak_) was lower than P^*ν*^_La.max_ (*p* < 0.0001, respectively). P^*ν*^_La.max_ and ^*ν*^_La.max_ (t_PCr−peak_) were highly correlated (*r* = 0.99; *R* ^*2*^ = 0.98). This correlation was higher than the relationship between P^*ν*^_La.max_ and ^*ν*^_La.max_ (t_PCr −3.5%_) (*r* = 0.87; *R* ^*2*^ = 0.77). ^*ν*^_La.max_ (t_PCr−peak_), P^*ν*^_La.max_, and ^*ν*^_La.max_ (t_PCr −3.5%_) were found to correlate with absolute W_mean_ and *W*_Gly_.

**Conclusion:** P^*ν*^_La.max_ as a modified calculation of ^*ν*^_La.max_ provides more detailed insights into the inter-individual differences in energy and glycolytic metabolism than ^*ν*^_La.max_ (t_PCr−peak_) and ^*ν*^_La.max_ (t_PCr −3.5%_). Because *W*_Oxi_ and *W*_PCr_ can differ remarkably between athletes, implementing their values in P^*ν*^_La.max_ can establish more optimized individual profiling for elite track cyclists.

## Introduction

Three human energy systems (phosphagen, glycolytic, and oxidative systems) are simultaneously used during different exercises (Beneke et al., 2002; Yang et al., 2020; Yang et al., 2022a; Yang et al., 2022b). However, their relative energy contribution, particularly the energy systems that are predominantly utilized at a given moment, depends critically on the exercise intensity and duration (Beneke et al., 2002; Yang et al., 2022b). Therefore, precise determinations of energetic contributions during specific sports are crucial for a better understanding of physiological responses to enhance exercise prescriptions (Julio et al., 2017; Franchini, 2020; Yang et al., 2022b).

Among various Olympic disciplines, there are sections such as football, handball, basketball, rugby, and track cycling (Hettinga et al., 2007; Wackerhage et al., 2022), in which maximal anaerobic power and capacity are essential to achieve a highly competitive performance. For instance, the anaerobic capacity of track cyclists is exploited during a 1,500-m Keirin race over approximately 90–120 s (Foster et al., 1993; Hettinga et al., 2007). A crucial factor that determines the winner of such races is particularly the final all-out sprint to the finish line (Fujii et al., 2018). Although a high aerobic performance is absolutely necessary, these athletes also require high anaerobic power (Hettinga et al., 2007; Fujii et al., 2018). The anaerobic performance of athletes (e.g., 100-m sprint) is proportional to their individual anaerobic energy capacity (Yang et al., 2020; Park et al., 2021; Yang et al., 2022b). The phosphagen system (adenosine triphosphate and phosphocreatine, ATP-PCr) ensures the highest metabolic power or energy flux rate, which is reflected by the rate of energy transfer per unit of time. Although the energy flux rate is very high, the general capacity is low because of limited substrate stores that lasts for only a few muscle contractions (Gastin, 2001; Robergs et al., 2004). The glycolytic system can re-synthesize ATP through the non-aerobic degradation of carbohydrates and supports the metabolic energy requirements during intense exercise. This system, when compared to the phosphagen system, is characterized by an intermediate energy flux rate and metabolic power because of the higher number of reactions and greater overall capacity from a large amount of stored carbohydrates (Gastin, 2001; Robergs et al., 2004; Franchini, 2023). In this regard, the Mader’s (1984) model proposed considering individual maximal glycolytic rates (^*ν*^_La.max_) to be utilized while determining the maximal glycolysis and power after an all-out sports-specific sprint test (Hauser et al., 2014; Quittmann et al., 2020; Quittmann et al., 2021; Wackerhage et al., 2022). Accordingly, athletes in sprint sports can maximally produce over 1.0 mmol lactate per liter per second (i.e., ≥1.0 mmol·L^−1^·s^−1^) (Wackerhage et al., 2022).

The formula of ^*ν*^_La.max_ (Mader, 1984) is considered the delta lactate concentration (∆La^−^) between maximal blood La^−^ accumulation after exercising and resting blood La^−^ levels are divided by the difference between the total exercise time (t_exer_) and phosphagen system–contributed time (t_PCr_). Here, t_PCr_ is assumed as the period in which no La^−^ formation (“fictitiously”) takes place, beginning from the start of the maximal power output until it is reduced by 3.5% (the point of −3.5% from the peak watt, t_PCr −3.5%_). Previous studies have used the t_PCr −3.5%_ definition without providing an in-depth explanation on why the reduced 3.5% time point of the maximal power output was determined as t_PCr_ (Hauser et al., 2014; Adam et al., 2015; Nitzsche et al., 2018; Quittmann et al., 2020; Quittmann et al., 2021). However, this assumption likely originates from the precision of power measurement of an early SRM cycle ergometer (Weber, 2003). Another t_PCr_ definition was determined as the time until peak watt (t_PCr−peak_) during the ^*ν*^_La.max_ test (Manunzio et al., 2016).

Indeed, the determination of ^*ν*^_La.max_ *via* these calculations may further have imprecisions with the involvement of oxidative energy metabolism, also in short time frames of exercising, when it is not separately subtracted from the total energy demand. Interestingly, previous studies have reported that oxidative energy metabolism is not considered in the ^*ν*^_La.max_ formula, which contributes to a total energy expenditure of approximately 10% during the 100-m sprint (Heck et al., 2003; Quittmann et al., 2020; Park et al., 2021).

Also, calculations of the Mader’s model include the oxidative contribution timespan and not subtracting the energy contribution of oxidative metabolism. When considering an approximate contribution of 10% during short sprints, a “pure” ^*ν*^_La.max_ determination during the maximal anaerobic cycling test cannot be precisely calculated. To determine ^*ν*^_La.max_, we minimize the oxidative contribution and lactate elimination during the aftermath of the test; its duration should be limited between 10 and 15 s (Heck et al., 2003; Quittmann et al., 2020). In this context, the PCr-La^−^-O_2_ method is yet another well-established methodology involving all three energy system contributions when calculating exercise-induced energy turnover (Beneke et al., 2004; de Moraes Bertuzzi et al., 2007; de Campos Mello et al., 2009; Hausen et al., 2017; Julio et al., 2017; Yang et al., 2018; Park et al., 2021; Yang et al., 2022b; Kaufmann et al., 2022). This method can easily be incorporated into the ^*ν*^_La.max_ formula as the time ratio of relative oxidative contribution (%) and then be used to calculate an optimized ^*ν*^_La.max_ (Beneke et al., 2004; de Moraes Bertuzzi et al., 2007; de Campos Mello et al., 2009; Campos et al., 2012; Hausen et al., 2017; Julio et al., 2017; Lopes-Silva et al., 2018; Yang et al., 2018; Franchini, 2020; Park et al., 2021; Yang et al., 2022b).

Therefore, this study compares three different ^*ν*^_La.max_ calculations based on the differences in determining the phosphagen-contributed time and incorporation of the oxidative energy system: first, ^*ν*^_La.max_ by using t_PCr −3.5%_; second, ^*ν*^_La.max_ by using t_PCr−peak_ determinations; and third, by subtracting the contribution of the oxidative energy metabolism during a 15-s maximal sprint cycling test. The latter is substituted with a modified pure ^*ν*^_La.max_ (P^*ν*^_La.max_) formula using the analysis of the PCr-La^−^-O_2_ method. Finally, the relationships among the different ^*ν*^_La.max_ calculations, mean power output, and glycolytic energy contribution are analyzed.

## Materials and methods

### Participants

With an effect size of 0.32, an alpha error probability of 0.05, and statistical power of 0.95, the sample size was calculated using the G*Power software, version 3.1.9.4 (Heinrich Heine University, Düsseldorf, Germany). The effect size was considered based on previous studies (Hauser et al., 2014; Manunzio et al., 2016; Fujii et al., 2018; Quittmann et al., 2020; Quittmann et al., 2021). Considering a 10% dropout rate, 30 male national-level track cyclists were enrolled in this study. All participants were track cyclists (main discipline: Keirin) who train for 18–20 h per week. They have been enrolled in the Korean national championships over the last 8 years. The anthropometric parameters of these participants are age: 30 ± 6 years; height: 177.30 ± 6.21 cm; body mass: 85.11 ± 9.89; and body fat: 19.16% ± 4.78%. The participants had rested for 2 h after lunchtime and performed a 15-s all-out sprint cycling test (ASCT). They were instructed not to take any coffee, neuro stimulants, or medication immediately before the test. Alcohol and nicotine was not consumed at least 24 h before the experiment. The Institutional Review Board of CHA University approved this study (No. 1044308-202206-HR-032-02). Study protocols followed the ethical standards in the Declaration of Helsinki. All participants had signed an informed consent form.

### Study procedure

**FIGURE 1:**
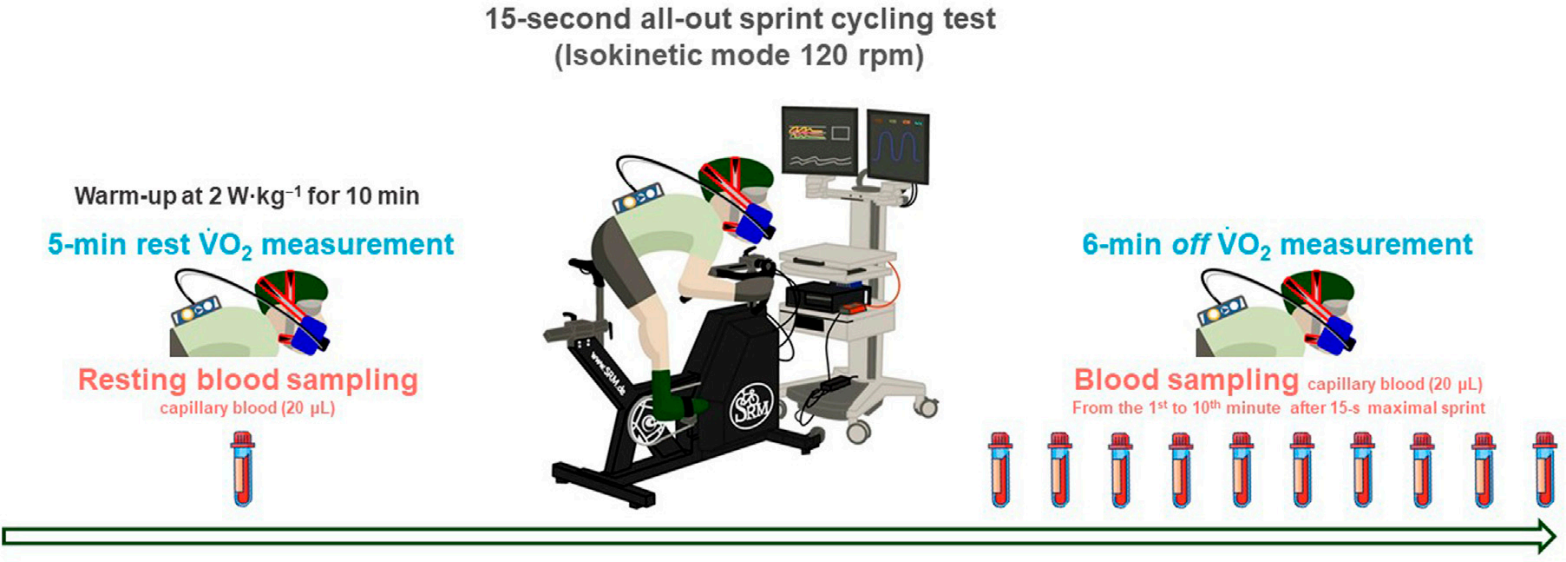
Study procedure. An initial warm-up was performed for 10 min at 2 W·kg^−1^. A further passive rest for 5 min followed. A 15-s all-out sprint cycling test was performed in the isokinetic mode set to a cadence of 120 rpm. Participants performed the test in the sitting position on the ergometer. Oxygen uptake at 5-min rest, and during as well as up to 6 min after 15-s all-out sprint cycling test was measured. Capillary blood was collected from the earlobe (20 μL) before and in minutes' interval (1st to 10th) after the 15-s all-out sprint cycling test. $\dot{V}$ O_2_, oxygen uptake.

All participants attended a single laboratory session. The test procedure was conducted and controlled in the same laboratory environment throughout testing (temperature: 23°C and relative humidity: 50%). The anthropometric parameters were measured with an eight-electrode segmental multi-frequency bioelectrical impedance analysis (20–100 kHz; InBody 270; InBody Co., Ltd., Seoul, Republic of Korea). Before the 15-s ASCT, the latest SRM ergometer (No. 2203, 04/2022, Schoberer Rad Messtechnik, GmbH, Jülich, Germany) was set up to fit each participant’s track bicycle position (i.e., crank length, saddle height, handle height, and saddle fore–aft position). An initial warm-up was performed for 10 min at 2 W·kg^−1^. They further rested passively in the sitting position on the ergometer was for 5 min (Figure 1). The 15-s ASCT was performed according to the manufacturer’s guidelines in an isokinetic mode set to a cadence of 120 rpm (Manunzio et al., 2016) in the ninth gear and with a 9.1 kg flywheel. The start and end of the 15-s ASCT were verbally announced by countdown. These settings enabled a reduction in the pedal force in a linear fashion during the initial acceleration phase and achieving a target pedaling rate of 120 rpm after ∼4 s (Manunzio et al., 2016; Dunst et al., 2023). The participants performed the test in the sitting position on the ergometer. They were verbally encouraged by the investigator to generate the maximum possible power output. Afterward, the absolute and relative peak and mean power outputs in watt and W·kg^−1^ were determined (W_peak_ and W_mean_, respectively) (Hauser et al., 2014; Manunzio et al., 2016; Quittmann et al., 2020; Quittmann et al., 2021). Oxygen uptake (resting oxygen uptake, $\dot{V}$ O_2rest_; average oxygen uptake during the 15-s ASCT, $\dot{V}$ O_2mean_; highest oxygen uptake during the 15-s ASCT, $\dot{V}$ O_2peak_; and fast component of excess oxygen consumption [EPOC_fast_], *off* $\dot{V}$ O_2_ kinetics) at 5-min rest, and during and after the 15-s ASCT (6 min) were measured using the breath-by-breath method with a portable gas analyzer (METAMAX 3B, Cortex Biophysik, Leipzig, Germany) that was placed on the back of the participants. The portable gas analyzer was calibrated with the calibration gas (15% O_2_ and 5% CO_2_; Cortex Biophysik, Leipzig, Germany), and the turbine volume transducer was calibrated using a 3-L syringe (Hans Rudolph, Kansas, United States). $\dot{V}$ O_2mean_ and $\dot{V}$ O_2peak_ were calculated as the average and highest values during the 15-s ASCT, respectively. Capillary blood was collected from the earlobe (20 μL) before and, in minute intervals (1st to 10th), after the 15-s ASCT to determine rest and maximal lactate concentrations (La_rest_ and La_max_; the maximal value of La^−^ among the 10 values) using an enzymatic–amperometric sensor chip system (Biosen C-line; EKF diagnostics sales, GmbH, Barleben, Germany).

### Calculations of energetic contributions (PCr-La^−^-O_2_ method)

Phosphagen (*W*_PCr_), glycolytic (*W*_Gly_), and oxidative (*W*_Oxi_) contributions in kilojoules, and their percentages were calculated by measuring $\dot{V}$ O_2_ and La^−^ (PCr-La^−^-O_2_) before, during, and after the 15-s ASCT (Julio et al., 2017; Yang et al., 2018; Yang et al., 2022b; Kaufmann et al., 2022).

*W*_PCr_ was calculated using $\dot{V}$ O_2_ after the 15-s ASCT and the fast component of excess post-exercise after the 15-s ASCT (Gastin, 2001; Beneke et al., 2004; Yang et al., 2022b). $\dot{V}$ O_2_ kinetics after the 15-s ASCT was fitted by mono-exponential and bi-exponential models using the OriginPro 2021 software (OriginLab Corp, Northampton, United States). The slow component of the bi-exponential model was negligible. Thus, $\dot{V}$ O_2_ data after the 15-s ASCT were fitted using a mono-exponential model, and *W*_PCr_ was obtained by calculating the integral of the exponential area (Beneke et al., 2004; Campos et al., 2012; Julio et al., 2017; Yang et al., 2018; Yang et al., 2022b; Kaufmann et al., 2022).

The *W*_Gly_ calculation was performed by determining La^−^ before and after the 15-s ASCT (La_rest_ and La_max_), assuming that the production of 1 mmol·L^−1^ was equivalent to 3 mL O_2_·kg^−1^ of body mass (di Prampero and Ferretti, 1999). The difference in La^−^ (∆La^−^) was determined by La^−^ after the 15-s ASCT minus La^−^ before the 15-s ASCT (Beneke et al., 2004; Campos et al., 2012; Yang et al., 2018; Yang et al., 2022b).

The *W*_Oxi_ contribution was calculated by subtracting $\dot{V}$ O_2rest_ from $\dot{V}$ O_2_ during the 15-s ASCT by the trapezoidal method in which the area under the curve was divided into sections and the sum of the trapezoid was used to calculate the integral (de Campos Mello et al., 2009; Yang et al., 2022b). The $\dot{V}$ O_2rest_ value was determined in the sitting position on the cycle ergometer in the last 30 s of the 5-min phase used as a reference (di Prampero and Ferretti, 1999; Beneke et al., 2002; Beneke et al., 2004; Julio et al., 2017; Yang et al., 2018; Kaufmann et al., 2022; Yang et al., 2022b). A caloric quotient of 20.92 kJ was used in the three energy system calculations (Gastin, 2001). The total energy expenditure was determined as the sum of the three energy systems in kilojoules (*W*_PCr_, *W*_Gly_, and *W*_Oxi_) (Campos et al., 2012). The relative contribution of each of the three energy systems was calculated in percentage and compared with the total energy expenditure.

### Different formulas of ^*ν*^_La.max_ calculations

^*ν*^_La.max_ (t_PCr −3.5%_) was calculated as ∆La^−^ between La_rest_ and La_max_, which was divided by the difference between the total exercise time (t_Exer_) and the −3.5% time point from the peak watt (t_PCr −3.5%_) (Mader, 1984; Weber, 2003) (Eq. 1):

La.maxvtPCr−3.5%mmol·L−1·s−1=Lamax−LaresttExer−tPCr−3.5%. (1)

Furthermore, only t_PCr_ was differently determined according to previous studies (Serresse et al., 1988; Beneke et al., 2002; Manunzio et al., 2016) (Eq. 2):

La.maxvtPCr−peak mmol·L−1·s−1=Lamax−LaresttExer−tPCr−peak. (2)

The accuracy of the latest SRM cycle ergometer (power meter in the science system) is reported to be ± 0.5–1% according to the manufacturer and previous studies (Bouillod et al., 2017; Nimmerichter et al., 2017; Montalvo-Pérez et al., 2021). Therefore, a representation of t_PCr−peak_ was determined as the time until W_peak_ during the 15-s ASCT because energy was influenced by the ATP-PCr system until peak power during the initial seconds of a maximal short-term exercise (Serresse et al., 1988; Beneke et al., 2002).

A modified pure ^*ν*^_La.max_ was calculated as ∆La^−^ divided by the difference between t_Exer_ and t_PCr−peak_ plus relative *W*_Oxi_ in percentage of the PCr-La^−^-O_2_ method during the 15-s ASCT. This percentage was converted to time in seconds (t_Oxi_) related to the total ASCT time (Eq. 3):

PLa.maxv mmol·L−1·s−1=Lamax−LaresttExer−tPCr−peak+tOxi. (3)

### Statistical analyses

All collected data were analyzed using GraphPad Prism 9.4.1. (GraphPad Prism Software Inc., La Jolla, CA, United States). The data of collected parameters are presented as mean ± standard deviation (SD). The normal distribution of all data was performed using the Shapiro−Wilk test. The contributions of the three energy systems and different ^*ν*^_La.max_ values were compared using a repeated-measures analysis of variance (ANOVA) with the Bonferroni *post hoc* test. The Mauchly’s sphericity test was used to determine whether the assumption of sphericity was being violated by the data. The Greenhouse−Geisser correction was required when necessary. A comparison between t_PCr −3.5%_ and t_PCr−peak_ was conducted using a paired *t*-test. The significance level was set at *p* < 0.05. The effect sizes (partial eta squared [ $\left.\eta_{p}^{2}\right]$ and Cohen’s [*d*]) were calculated for the main effect. The thresholds for small, medium, and large effects were 0.01, 0.06, and 0.14 for partial eta squared [ $\eta_{p}^{2}$ ] and 0.2, 0.5, and 0.8 for Cohen’s [*d*], respectively (Fritz et al., 2012). The relationships were analyzed with a two-tailed Pearson’s correlation [^*ν*^_La.max_ (t_PCr −3.5%_) *vs.* P^*ν*^_La.max_, ^*ν*^_La.max_ (t_PCr−peak_) *vs.* P^*ν*^_La.max_, and W_mean_ and *W*_Gly_ *vs.* different ^*ν*^_La.max_]. The Bland–Altman plots were determined to analyze the bias, difference, average, and 95% limit agreements among the ^*ν*^_La.max_ (t_PCr −3.5%_), the ^*ν*^_La.max_ (t_PCr−peak_), and the P^*ν*^_La.max_ (Bland and Altman, 1995).

## Results

### Energetic contributions during 15-s ASCT

**FIGURE 2:**
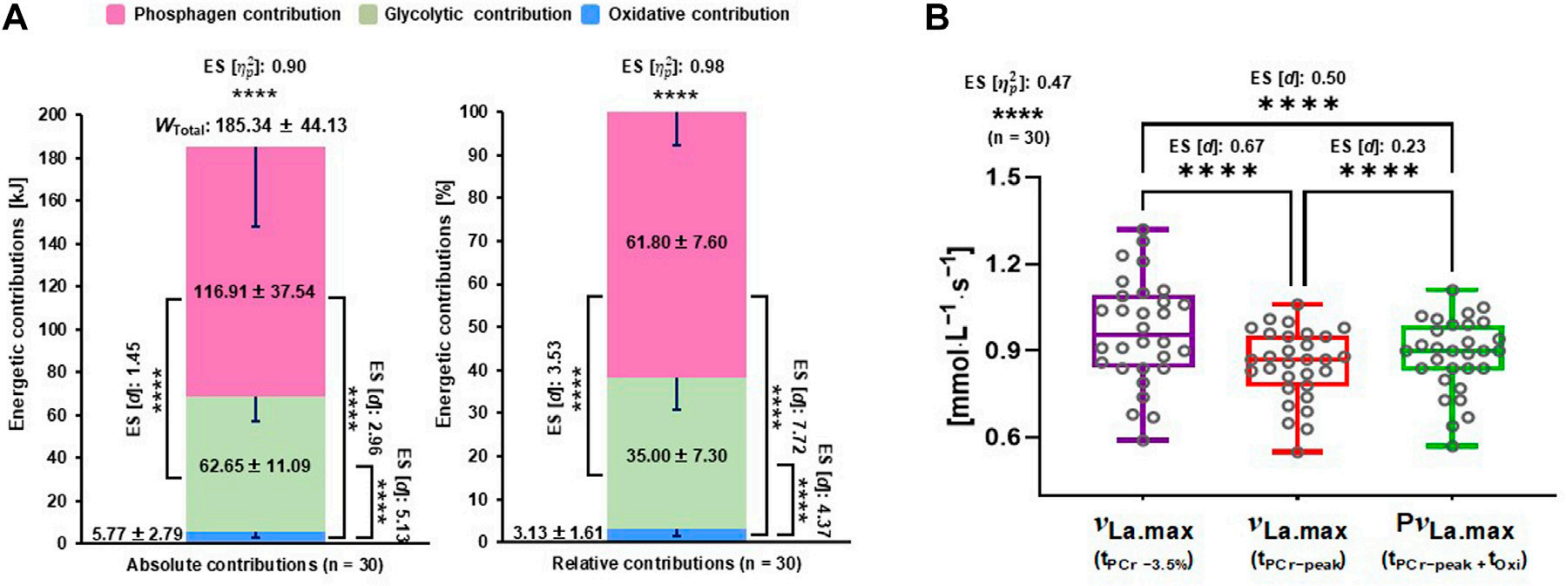
Energetic contributions and different calculations of ^*ν*^_La.max_. **(A)** Comparisons of three energy contributions in kilojoules and percentage, and **(B)** comparisons of three different ^*ν*^_La.max_ calculations. ES, effect sizes; ^*ν*^_La.max_, maximal rate of lactate accumulation; P^*ν*^_La.max_, pure maximal rate of lactate accumulation; t_Oxi_, time of oxidative energy system contribution; t_PCr−peak_, phosphagen energy system–contributed time until the peak power output; t_PCr −3.5%_, phosphagen energy system contributed −3.5% time point from the peak power output; *W*_Total_, total energy demand. Significant difference; ****p < .0001.

The repeated-measures ANOVA indicated significant differences in energetic contributions in kilojoules and their percentages (*p* < 0.0001, effect size [ $\eta_{p}^{2}$ ]: 0.90; *p* < 0.0001, [ $\eta_{p}^{2}$ ]: 0.98, respectively). The value of *W*_PCr_ was significantly higher than that of *W*_Gly_ and *W*_Oxi_ in kilojoules as well as in percentage (*p* < 0.0001, [*d*]: 1.45, 2.96, and 5.13 in kJ; *p* < 0.0001, [*d*]: 3.53, 7.72, and 4.37 in percentage, respectively) (Figure 2A).

### Physiological parameters and relationships between different calculations of ^*ν*^_La.max_

The time duration (s) of the ^*ν*^_La.max_-related t_PCr −3.5%_ was significantly higher than t_PCr−peak_ (*p* < 0.0001, [*d*]: 1.42; Table 1). The data of the other physiological parameters of anaerobic performance are presented as mean ± SD in Table 1.

**TABLE 1:** Anaerobic performances and physiological parameters during a 15-s all-out sprint cycling test.

Parameter	Eq. 1 (t_PCr −3.5%_)	Eq. 2 (t_PCr−peak_)	Eq. 3 (t_PCr−peak +_ t_Oxi_)
^*ν*^_La.max_ [mmol∙L^−1^∙s^−1^]	0.97 ± 0.18	0.85 ± 0.12^a****^	0.88 ± 0.13^a,b****^
t_PCr_ [s]	3.28 ± 1.08	1.75 ± 0.59^a****^	
t_Oxi_ [s]			0.49 ± 0.25
t_Exer_ [s]	15.48 ± 0.16		
W_peak_ [W]	1496.13 ± 146.00		
W_peak_ [W∙kg^−1^]	17.84 ± 1.73		
W_mean_ [W]	1260.43 ± 118.77		
W_mean_ [W∙kg^−1^]	15.02 ± 1.31		
La_rest_ [mmol∙L^−1^]	1.01 ± 0.27		
La_max_ [mmol∙L^−1^]	12.74 ± 1.74		
ΔLa^−^ [mmol∙L^−1^]	11.74 ± 1.63		
$\dot{V}$ O_2peak_ [mL∙kg^−1^∙min^−1^]	26.47 ± 7.82		
$\dot{V}$ O_2mean_ [mL∙kg^−1^∙min^−1^]	21.25 ± 5.94		

Data are present as mean ± standard deviation (*n* = 30). Eq., equation of the maximal rate of lactate accumulation; La_rest_, lactate concentration at rest; La_max_, maximal lactate concentration; ΔLa^−^, difference between La_rest_ and La_max_; t_PCr_, contributed time of the phosphagen system; t_Oxi_, contributed time of the oxidative system; t_Exer_, total exercise duration; $\dot{V}$ O_2peak_, highest oxygen uptake; $\dot{V}$ O_2mean_, mean oxygen uptake; ^ν^_La.max_, maximal rate of lactate accumulation; W_mean_, mean power output in watt; W_peak_, highest power output in watt. a *vs.* Eq. 1; b *vs.* Eq. 2; ****p < 0.0001.

The repeated-measures ANOVA showed significant differences of the three ^*ν*^_La.max_ values (*p* < 0.0001, [ $\eta_{p}^{2}$ ]: 0.47). The level of ^*ν*^_La.max_ (t_PCr −3.5%_) was significantly higher than that of ^*ν*^_La.max_ (t_PCr−peak_) and P^*ν*^_La.max_ (*p* < 0.0001, [*d*]: 0.67 and 0.50, respectively). A significantly lower value of ^*ν*^_La.max_ (t_PCr−peak_) than that of P^*ν*^_La.max_ (*p* < 0.0001, [*d*]: 0.23) was observed (Table 1; Figure 2B).

**FIGURE 3:**
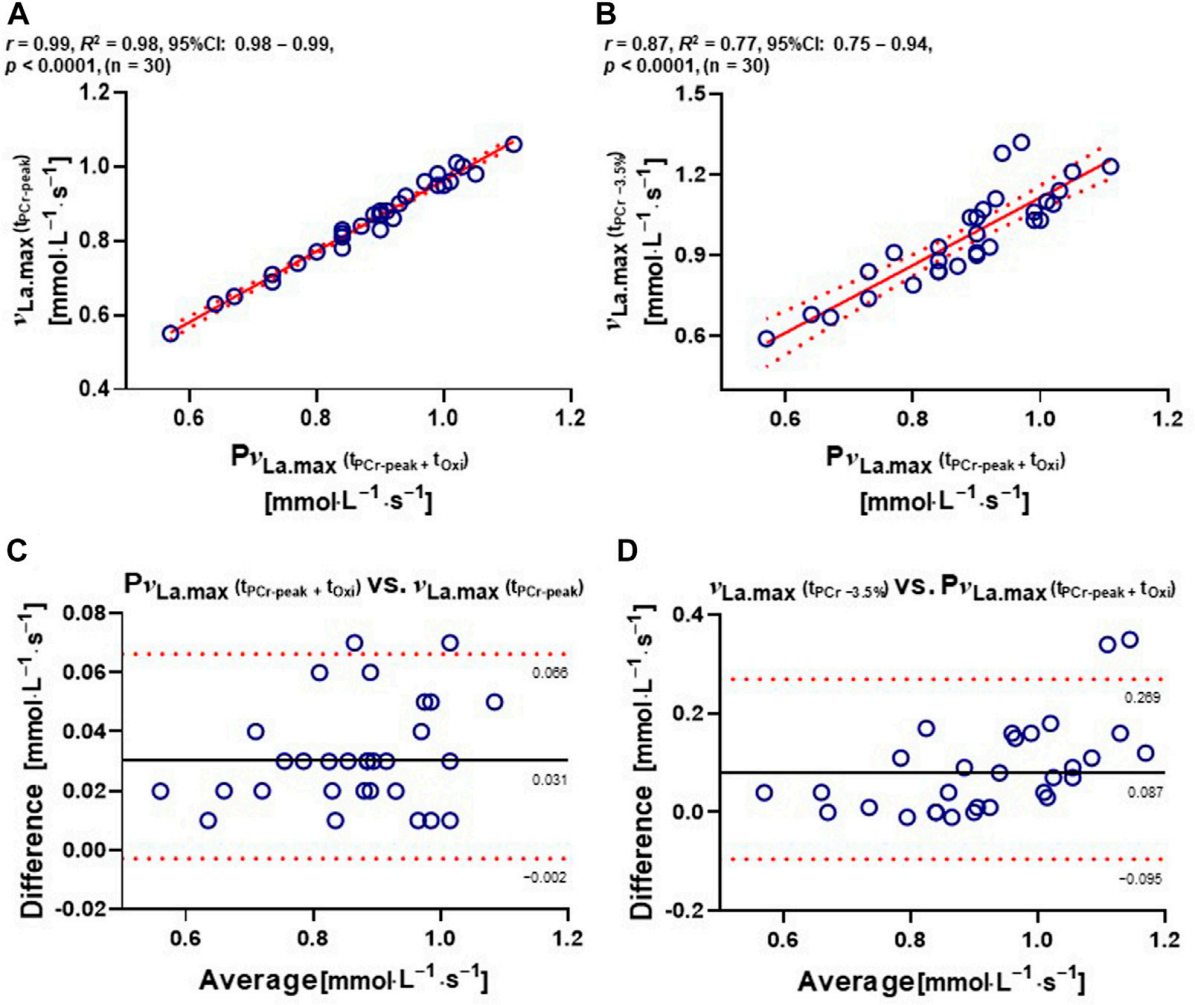
Relationships and Bland–Altman plots between different calculations of ^*ν*^_La.max_. **(A)** Two-tailed Pearson’s correlation between P^*ν*^_La.max_ and ^*ν*^_La.max_ (t_PCr−peak_). **(B)** Two-tailed Pearson’s correlation between P^*ν*^_La.max_ and ^*ν*^_La.max_ (t_PCr −3.5%_). **(C and D)** Bland–Altman plots of differences between P^*ν*^_La.max_ and ^*ν*^_La.max_ (t_PCr−peak_) and between ^*ν*^_La.max_ (t_PCr −3.5%_) and P^*ν*^_La.max_. The dashed line represents bias. The dotted line represents 95% limit of agreements. CI, confidence interval; ES, effect sizes; ^*ν*^_La.max_, maximal rate of lactate accumulation; P^*ν*^_La.max_, pure maximal rate of lactate accumulation; t_Oxi_, time of oxidative energy system contribution; t_PCr−peak_, phosphagen energy system–contributed time until the peak power output; t_PCr −3.5%_, phosphagen energy system contributed −3.5% time point from the peak power output. Significant difference; ****p < .0001.

A very strong correlation was found between P^*ν*^_La.max_ and ^*ν*^_La.max_ (t_PCr−peak_) (*r* = 0.99; *R* ^2^ = 0.98; 95% confidence interval [CI]: 0.98–0.99; *p* < 0.0001) (Figure 3A). A further high positive correlation between P^*ν*^_La.max_ and ^*ν*^_La.max_ (t_PCr −3.5%_) was observed (*r* = 0.87; *R* ^2^ = 0.77; 95% CI: 0.75–0.94; *p* < 0.0001) (Figure 3B). The Bland–Altman analysis showed that the bias between P^*ν*^_La.max_ and ^*ν*^_La.max_ (t_PCr−peak_) calculations was 0.031 ± 0.017. Another bias between ^*ν*^_La.max_ (t_PCr −3.5%_) and P^*ν*^_La.max_ calculations was 0.087 ± 0.092. P^*ν*^_La.max_ and ^*ν*^_La.max_ (t_PCr−peak_) calculations indicated a smaller 95% limit of agreement (−0.002 to 0.066) followed by ^*ν*^_La.max_ (t_PCr −3.5%_) and P^*ν*^_La.max_ (−0.095 to 0.269) (Figures 3C, D).

### Relationships between anaerobic performance and different ^*ν*^_La.max_

**FIGURE 4:**
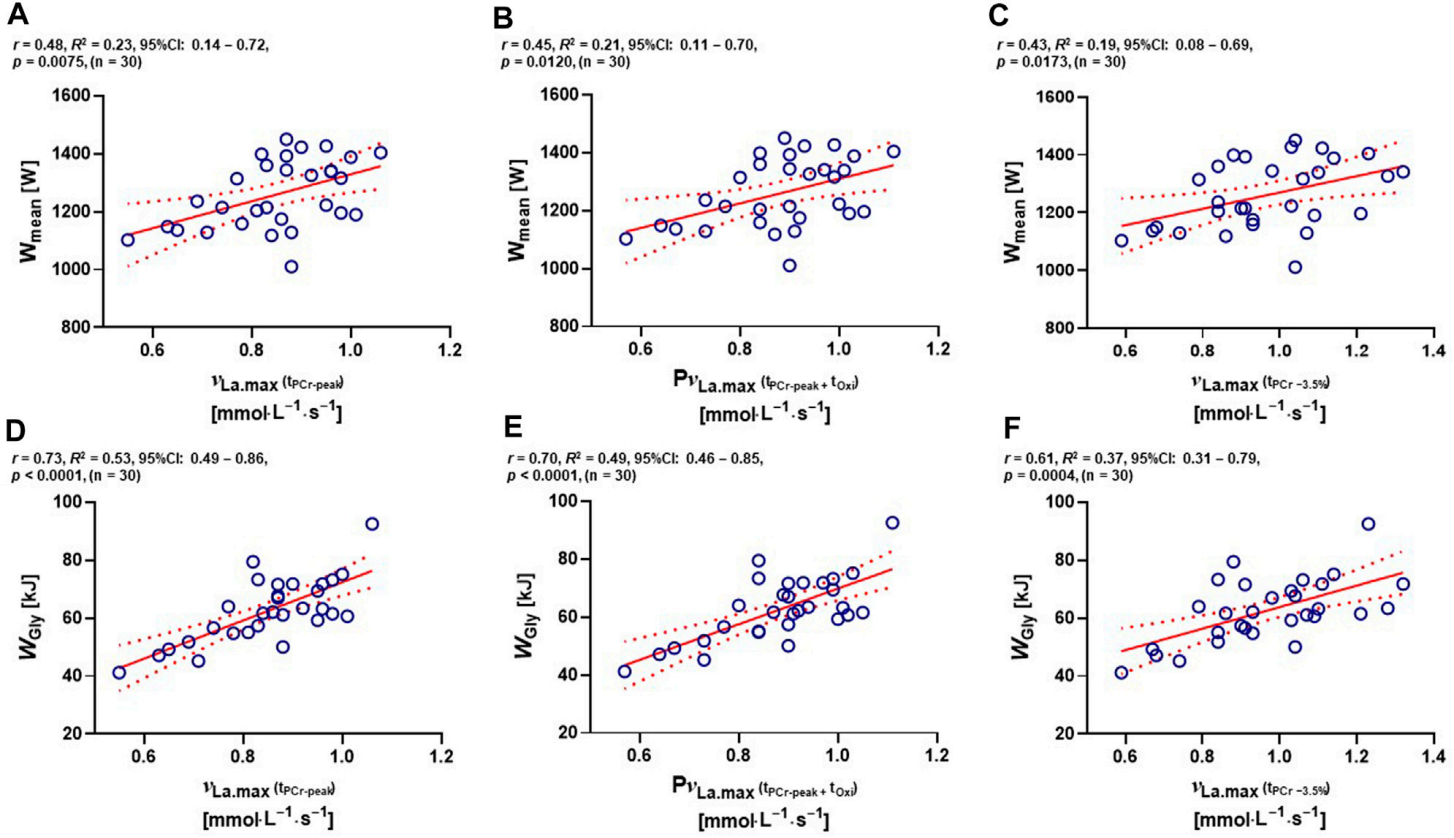
Two-tailed Pearson’s correlations **(A)** between ^*ν*^_La.max_ (t_PCr−peak_) and W_mean_, **(B)** between P^*ν*^_La.max_ and absolute W_mean_, **(C)** between ^*ν*^_La.max_ (t_PCr −3.5%_) and absolute W_mean_, **(D)** between ^*ν*^_La.max_ (t_PCr−peak_) and absolute *W*_Gly_, **(E)** between P^*ν*^_La.max_ and absolute *W*_Gly_, and **(F)** between ^*ν*^_La.max_ (t_PCr −3.5%_) and absolute *W*_Gly_. CI, confidence interval; ^*ν*^_La.max_, maximal rate of lactate accumulation; P^*ν*^_La.max_, pure maximal rate of lactate accumulation; t_Oxi_, time of oxidative energy system contribution; t_PCr−peak_, phosphagen energy system contribution time until peak power output; t_PCr −3.5%_, phosphagen energy system contribution at −3.5% time point from peak power output; *W*_Gly_, absolute glycolytic energy contribution in kilohoules; W_mean_, absolute mean power output in watt.

Low positive correlations between ^*ν*^_La.max_ (t_PCr−peak_) and absolute W_mean_, P^*ν*^_La.max_ and absolute W_mean_, and ^*ν*^_La.max_ (t_PCr −3.5%_) and absolute W_mean_ were observed (*r* = 0.48, *R* ^2^ = 0.23, 95% CI: 0.14–0.72, and *p* = 0.0075; *r* = 0.45, *R* ^2^ = 0.21, 95% CI: 0.11–0.70, and *p* = 0.0120; *r* = 0.43, *R* ^2^ = 0.19, 95% CI: 0.08–0.69, and *p* = 0.0173, respectively) (Figures 4A−C). Furthermore, moderate to high positive correlations between ^*ν*^_La.max_ (t_PCr−peak_) and *W*_Gly_, P^*ν*^_La.max_ and *W*_Gly_, and ^*ν*^_La.max_ (t_PCr −3.5%_) and *W*_Gly_ in kilojoules were found (*r* = 0.73, *R* ^2^ = 0.53, 95% CI: 0.49–0.86, and *p* < 0.0001; *r* = 0.70, *R* ^2^ = 0.49, 95% CI: 0.46–0.85, and *p* < 0.0001; *r* = 0.61, *R* ^2^ = 0.37, 95% CI: 0.31–0.79, and *p* = 0.0004, respectively) (Figures 4D−F).

## Discussion

In previous studies, calculations of ^*ν*^_La.max_ for the maximal glycolytic capacity after the 15-s ASCT were limited because t_PCr_ and aerobic energy contribution were not considered. To the best of our knowledge, this study is the first to integrate the oxidative energy contribution and contributions of the three energy systems during the 15-s ASCT *via* a modified P^*ν*^_La.max_ calculation. This modified P^*ν*^_La.max_ was compared with previous ^*ν*^_La.max_ calculations for glycolytic performance and their correlations were determined.

The main findings of this study confirm that phosphagen and glycolytic systems are predominantly utilized, while the oxidative energy metabolism contributes to 3.13% ± 1.61% during the 15-s ASCT. The t_PCr−peak_ and t_Oxi_- subtracted P^*ν*^_La.max_ values were lower than ^*ν*^_La.max_ (t_PCr −3.5%_) but higher than ^*ν*^_La.max_ (t_PCr−peak_) values. Moreover, very strong and high correlations between P^*ν*^_La.max_ and ^*ν*^_La.max_ (t_PCr−peak_) and between P^*ν*^_La.max_ and ^*ν*^_La.max_ (t_PCr −3.5%_) were found. Three different ^*ν*^_La.max_ values were positively associated with absolute W_mean_ and *W*_Gly_ values.

The phosphagen energy system has the highest absolute energetic contribution during the 15-s ASCT, followed by glycolytic and oxidative energy metabolism, thus being in agreement with the results of a previous study by Park et al. (2021). In this study, all three energy systems were analyzed using the same PCr-La^−^-O_2_ method during a 100-m sprint and the oxidative energy system contributed over 10% here to the total energy requirements. In comparison to this outcome, the oxidative contribution in the present study was lower (∼3%) during the 15-s ASCT (Figure 2A). These different results might be because 100-m sprint and sprint cycling are differently characterized by maximal anaerobic performances, which include different movements of the upper extremities. Previous studies have reported that smaller muscles such as those in the upper extremities do not significantly contribute to total lactate accumulation (Julio et al., 2017; Yang et al., 2022b). Therefore, glycolytic energy contribution during the 15-s ASCT likely had a higher percentage (35% ± 7%) than during a 100-m sprint (∼25 ± 7%) as obtained in a previous study outcome (Park et al., 2021), which in turn reduced the relative oxidative contribution.

Regarding the different calculations of ^*ν*^_La.max_, the level of ^*ν*^_La.max_ (t_PCr −3.5%_) showed significantly higher values than ^*ν*^_La.max_ (t_PCr−peak_) and P^*ν*^_La.max_ (Figure 2B) because the t_PCr −3.5%_ value calculated was 3.2 ± 1.0 s during the 15-s ASCT (Table 1) such that the denominator (∆time) of the formula was smaller than the others. Due to the same reason, in including t_PCr−peak_ + t_Oxi_, P^*ν*^_La.max_ showed higher values than ^*ν*^_La.max_ (t_PCr−peak_). According to our results, the calculation of ^*ν*^_La.max_ (t_PCr −3.5%_) was unsuitable to determine the glycolytic capacity or ^*ν*^_La.max_ because the determined t_PCr_ time of −3.5% from W_peak_ was based on an error of the early SRM cycle ergometer (Weber, 2003). On the other hand, previous researchers suggested no lactate production during t_PCr_ which was incorrectly (fictitiously) assumed (Heck et al., 2003; Hauser et al., 2014; Adam et al., 2015; Nitzsche et al., 2018; Quittmann et al., 2020; Quittmann et al., 2021). In this regard, it is well known that all three energy systems start to work simultaneously and lactate production occurs independently of O_2_ availability such as under anoxic, hypoxic, and normoxic conditions (Gastin, 2001; Philp et al., 2005; Brooks, 2018; Yang et al., 2020; Brooks et al., 2022; Yang et al., 2022b). La^−^ might be accumulated at a relatively low level during the initial seconds of the 15-s ASCT because ATP-PCr is a dominant energy contribution until the achievement of W_peak_ (Serresse et al., 1988; Beneke et al., 2002; Park et al., 2021). Moreover, Heck et al. (2003) have suggested that aerobic power and contribution are of minor significance during maximal short-term exercise testing. However, this interpretation is based on the results of a computer simulation that originated from systems biology (Wackerhage et al., 2022). The calculated ^*ν*^_La.max_ values with and without t_Oxi_ show significant differences in our study's outcomes (Table 1; Figure 2B). Therefore, oxidative metabolism should be considered when aiming to analyze a pure maximal glycolytic rate. Considering the abovementioned factors, the accuracies of ^*ν*^_La.max_ (t_PCr−peak_) and P^*ν*^_La.max_ calculations seem to be higher because the durations of t_PCr−peak_ and t_Oxi_ are additionally involved in calculating formulas by subtracting them from the total exercise time. Accordingly, P^*ν*^_La.max_ was very strongly correlated with ^*ν*^_La.max_ (t_PCr−peak_), and this correlation was higher than that of P^*ν*^_La.max_ with ^*ν*^_La.max_ (t_PCr −3.5%_). The Bland–Altman plot between P^*ν*^_La.max_ and ^*ν*^_La.max_ (t_PCr−peak_) also showed a smaller limit of agreement than the limit of agreement between ^*ν*^_La.max_ (t_PCr −3.5%_) and P^*ν*^_La.max_ (Figures 3C, D). Furthermore, P^*ν*^_La.max_ and ^*ν*^_La.max_ (t_PCr−peak_) were associated with absolute W_mean_ and *W*_Gly_. Such associations were higher than those of ^*ν*^_La.max_ (t_PCr −3.5%_), with absolute W_mean_ and *W*_Gly_ (Figures 4A−F). These results indicate that P^*ν*^_La.max_ and ^*ν*^_La.max_ (t_PCr−peak_) are better calculating methods for glycolytic capacity/power.

To determine the anaerobic performance, the 30-s Wingate anaerobic test is often used in related studies (Bar-Or, 1987; Gastin, 2001; Zupan et al., 2009; Jaafar et al., 2014). However, this time frame will likely increase the oxidative contribution to the total energy demand and may also lead to an inhibition of phosphofructokinase activity because of accumulated hydrogen ions from increased ATP hydrolysis and reduced pH-levels (Gastin, 2001; Heck et al., 2003; Robergs et al., 2004; Yang et al., 2022b). This increased oxidative contribution may then reduce lactate accumulation and increase lactate elimination, which results in a reduced likelihood of measuring the maximal lactate accumulation (Heck et al., 2003; Yang et al., 2020; Yang et al., 2022b; Wackerhage et al., 2022). Thus, the ^*ν*^_La.max_ determination combining the 15-s ASCT with the P^*ν*^_La.max_ formula using the PCr-La^−^-O_2_ method is more suitable to limit the activation of oxidative metabolism as much as possible in decreasing intracellular lactate oxidation (Heck et al., 2003; Wackerhage et al., 2022).

In the current study, the levels of ^*ν*^_La.max_ (t_PCr −3.5%_) were higher than those of P^*ν*^_La.max_ and ^*ν*^_La.max_ (t_PCr−peak_), while a very strong relationship between P^*ν*^_La.max_ and ^*ν*^_La.max_ (t_PCr−peak_) was observed, one that was better than the relationship between P^*ν*^_La.max_ and ^*ν*^_La.max_ (t_PCr −3.5%_). Therefore, the calculation of P^*ν*^_La.max_ using the analysis of energetic contributions should be considered in scientific research to precisely analyze the maximal rate of lactate accumulation and anaerobic capacity/power. In practical diagnostics, the calculation of ^*ν*^_La.max_ (t_PCr−peak_) may be useful without a complicated analysis of energetic contributions or if it is not possible to be available for gas analyzer equipment eventually. Otherwise, the use of ^*ν*^_La.max_ (t_PCr −3.5%_) calculation is only suggested if the early version of the SRM cycle ergometer is available to perform the 15-s ASCT.

This study successfully identified the differences of three possible ^*ν*^_La.max_ calculations during the 15-s ASCT. However, the current study has some limitations. Because a spiroergometry system processing a dynamic micro-mixing chamber mode was not available during the test, the $\dot{V}$ O_2_ data were measured with the breath-by-breath method using a portable gas analyzer during the 15-s ASCT, which was converted to calculate *W*_Oxi_. This measurement mode during a maximal effort exercise has some errors (2–4%, approximately). Thus, the outcomes might be influenced by this factor. Moreover, although the t_PCr−peak_ determination is the physiologically acceptable method using the peak power output in sports science, the direct measuring of t_PCr_ is very difficult or impossible. It has never been measured using biomarkers/sensors such as the isotopic tracers or fluorescent protein sensors during a maximal short-term effort in humans. Actual direct measurements for t_PCr_ and exact metabolic contributions have to be determined in future studies. Further studies might also have to develop sport-specific P^*ν*^_La.max_ testing in other high-intensity intermittent sports disciplines such as football, handball, basketball, rugby, and combat sports as anaerobic performance profiling.

Regarding the possible gender differences in energetic contributions, Yang et al. (2022a) recently calculated the energy system contributions during increasing exercise intensities. The results found different *W*_PCr_ and *W*_Gly_ levels between males and females that were attributed to the differences in skeletal muscle mass and all fiber types. Therefore, there may be a need for further research into gender differences in the P^*ν*^_La.max_ calculation. Furthermore, the distribution of muscle fiber types might be considered when interpreting high or low ^*ν*^_La.max_ values in individual athletes with higher muscle mass and more glycolytic type IIA and IIX fibers who show generally higher lactate production during aerobic and anaerobic exercises (Ivy et al., 1980; Wackerhage et al., 2022). Future studies must show to what extent ^*ν*^_La.max_ might shift during a training year in track athletes with a different ^*ν*^_La.max_.

Moreover, there are currently no clear data available about which specific individual training sessions are helpful in improving ^*ν*^_La.max_. A recent study by Schünemann et al. (2023) suggests that sprint training, resistance training, and hypoxic training may increase glycolytic capacity and anaerobic performance. However, direct interventional approaches related to improving maximal glycolytic metabolism as P^*ν*^_La.max_ should be investigated further.

## Conclusion

The findings of the current study indicate that a modified calculation of P^*ν*^_La.max_ with the additional incorporation of further energetic contributions is precision enhancing and therefore valuable for determining the maximal glycolytic rate or ^*ν*^_La.max_. Our modified calculation therefore allows for a more detailed input of individual characteristics of energy metabolism to becoming an increasingly popular method for determining differences in maximal rates of lactate accumulation. In light of inter-individual differences in maximal force generation, phosphagen levels, and oxidative capacity, our method allows for compensating this interaction during ^*ν*^_La.max_ measurements when compared to previous methods. According to our results, the calculated formula of P^*ν*^_La.max_ should be used for scientific research in exercise sciences to determine the maximal anaerobic capacity/power more precisely and with the exclusion of the aerobic component. This is supported by a small limit of agreement (a small variation) and very high correlation with ^*ν*^_La.max_ (t_PCr−peak_). Furthermore, the calculation of ^*ν*^_La.max_ (t_PCr−peak_) might be recommended for a faster analysis of the practical approach in the field.

## Data Availability

The original contributions presented in the study are included in the article/Supplementary Material; further inquiries can be directed to the corresponding authors.
