# Corrigendum: A modified formula using energy system contributions to calculate pure maximal rate of lactate accumulation during a maximal sprint cycling test

**DOI:** 10.3389/fphys.2023.1297684

**Published:** 2023-12-11

**Authors:** Woo-Hwi Yang, So-Young Park, Taenam Kim, Hyung-Jin Jeon, Oliver Heine, Sebastian Gehlert

**Affiliations:** ^1^ Graduate School of Sports Medicine, CHA University, Pocheon-si, Gyeonggi-do, Republic of Korea; ^2^ Department of Medicine, General Graduate School, CHA University, Pocheon-si, Gyeonggi-do, Republic of Korea; ^3^ Olympic Base Center Rhineland, Cologne, Germany; ^4^ Department for Biosciences of Sports, Institute of Sports Science, University of Hildesheim, Hildesheim, Germany; ^5^ Institute of Cardiovascular Research and Sports Medicine, German Sport University Cologne, Cologne, Germany

**Keywords:** anaerobic performance, diagnostics, glycolytic metabolism, lactate, anaerobic power output

In the published article, there was an error in **Results,** *Physiological parameters and relationships between different calculations,* paragraph 2. A result was mistakenly explained as an incorrect parameter.

This sentence previously stated:

“A significantly lower value of ^*ν*^_La.max_ (t_PCr −3.5%_) than that of P^*ν*^_La.max_ (*p* < 0.0001, [*d*]: 0.23) was observed”. The corrected sentence should read as follows: “A significantly lower value of ^*ν*^_La.max_ (t_PCr−peak_) than that of P^*ν*^_La.max_ (*p* < 0.0001, [*d*]: 0.23) was observed.”

The authors apologize for this error and state that this does not change the scientific conclusions of the article in any way. The original article has been updated.

